# Diagnostic accuracy of handheld ultrasound, performed in community and hospital settings at 36 weeks to determine fetal presentation: the Sono-breech study protocol

**DOI:** 10.1136/bmjopen-2026-122247

**Published:** 2026-09-23

**Authors:** Jessica Thomas, Eleri Owen-Jones, Mariya Kovalenko, Anna Clark, Sana Usman, Suzanne Beattie-Jones, Danielle Thornton, Jayanta Banerjee, Neelam Heera-Shergill, Mandie Scamell, Cassandra Yuill, Julia Townson, Rachael Hunter, Jennifer Condie, Rebecca Playle, Amarnath Bhide, Christoph Lees

**Affiliations:** 1 Department of Metabolism, Digestion and Reproduction, Imperial College London, London, UK; 2 Cardiff University Centre for Trials Research, Cardiff, UK; 3 Imperial College Healthcare NHS Trust, London, UK; 4 Women’s Health Research Centre, Imperial College London, London, UK; 5 Department of Neonatology, Imperial College Healthcare NHS Trust, London, UK; 6 City St George’s University of London, London, UK; 7 Centre for Maternal and Child Health, City St George’s University of London, London, UK; 8 Cardiff University, Cardiff, UK; 9 Research Department of Primary Care and Population Health, University College London, London, UK; 10 Medical Statistics, Cardiff University Centre for Trials Research, Cardiff, UK; 11 City St George’s, University of London, London, UK

**Keywords:** Ultrasonography, Pregnancy, OBSTETRICS, Midwifery

## Abstract

**Abstract:**

**Introduction:**

Around 4% of babies are in breech presentation at the end of pregnancy but up to 43% of these are missed by abdominal palpation alone. Since women in the UK without additional risk factors do not routinely receive a scan in the third trimester, around 1%–2% of women present with undiagnosed breech presentation in labour, which is associated with poorer outcomes for both mothers and babies. Research suggests a point-of-care ultrasound (PoCUS) scan in late pregnancy could improve the rate of breech presentation detection. The Sono-breech study aims to determine the diagnostic accuracy and acceptability of handheld scans performed by midwives for fetal presentation at 36 weeks and how cost-effective this would be to implement in routine antenatal care in the National Health Service (NHS).

**Methods and analysis:**

Prior to delivering the study, midwives receive training for the detection of presentation and fetal heart rate using handheld ultrasound equipment.

The study will take place in at least 10 NHS Trusts across England selected to ensure a diverse participant population and to include under-researched settings. Between 35^+0^ and 36^+6^ weeks’ gestation, women who consent to the study will receive a scan for fetal presentation performed by a midwife using a handheld PoCUS device alongside routine antenatal checks, including palpation. Before the end of the next day, they will receive a conventional ultrasound scan performed by a practitioner deemed already competent at obstetric scanning. This scan will be considered the gold standard for presentation. All results will be recorded in the Sono-breech REDCap database. Women with babies in non-cephalic presentations will be referred to their local NHS Trust pathway for further management. Diagnostic accuracy (sensitivity and specificity) will be computed from the comparison between findings at the midwife-performed scan and the conventional ultrasound scan.

Birth outcome data will be collected from the participants’ medical notes after they have given birth. Aggregate birth data regarding the rate of undiagnosed non-cephalic births will be collected from all women who deliver at each site.

Midwives who take part in the study will be invited to participate in focus groups or interviews about the implementation of the scan into routine care. Non-participant observation will take place during midwife training sessions and antenatal clinics in specific participating trusts.

6–12 weeks after birth, some women will be invited to take part in an interview to explore their experience with the handheld scan.

6 weeks after birth, all women will be sent two questionnaires by email. These questionnaires will be used in combination with the birth outcome data and NHS tariffs to calculate the cost-effectiveness of introducing the handheld scan into routine antenatal care in the NHS.

Study registration ISRCTN11748052.

**Ethics and dissemination:**

The study has obtained approval from the West Midlands–South Birmingham Research Ethics Committee and was approved by the Health Research Authority and Health and Care Research Wales. Publication will be in line with the National Institute of Health and Care Research (NIHR) Open Access policy.

**STRENGTHS AND LIMITATIONS OF THIS STUDY**

- The study is undertaken in both hospital and community settings, increasing the relevance and applicability of the findings to routine antenatal care.
- The mixed-methods design, incorporating qualitative and health economic evaluations alongside the diagnostic accuracy study, enables assessment of the acceptability and feasibility of handheld point-of-care ultrasound.
- Use of conventional ultrasound as the reference standard for all participants minimises verification bias and provides robust estimates of diagnostic accuracy.
- Although the interval between the handheld ultrasound scan and conventional scan was selected to balance feasibility with minimising changes in fetal presentation, spontaneous version of the fetus between scans may still occur, is impossible to control for and could influence estimates of diagnostic accuracy.

## Introduction

 Around 4% of babies are in breech presentation at the end of pregnancy. In the UK, women without additional risk factors do not receive a scan in the third trimester; therefore, fetal presentation is primarily determined by abdominal palpation. Up to 43% of breech presentations may be missed by palpation,^1^ meaning breech presentation may not be diagnosed until the onset of or during labour. At this point, turning the baby by external cephalic version is usually not possible and is only attempted when the breech is not engaged (eg, in a footling breech).^2^ This means there is little time for the woman to fully consider the risks and benefits of having an emergency caesarean section or vaginal breech birth, and women often feel they have little choice.^3^ This situation is complicated further by midwives reporting they feel inadequately trained in managing vaginal breech delivery,^4^ and the Royal College of Obstetricians and Gynaecologists acknowledges a lack of experience and loss of skills in delivering breech babies vaginally in the UK.^5^

Vaginal breech birth increases the risk of fetal injury, low Apgar scores and perinatal morbidity and mortality,^6–8^ and Caesarean section increases the risk of maternal morbidity^8^ as well as increasing the risk of complications in future pregnancies. 12% of major litigation claims related to cerebral palsy between 2012 and 2016 involved breech birth, the majority of which were undiagnosed breech presentations in labour,^9^ and there have been damages settlements of up to £37 million for a baby injured during an undiagnosed breech birth.^10^ Furthermore, a birth that significantly deviates from the birth plan is associated with an increased risk of perinatal post-traumatic stress disorder.^11^ Therefore, diagnosing breech presentation before the onset of labour allows for a safe, realistic birth plan to be made.

Recently, there has been an increase in the use of point-of-care ultrasound (PoCUS) across a number of medical specialties, including in-hospital settings such as emergency medicine^12^ and cardiology,^13^ and out-of-hospital settings such as critical care transport.^14^ PoCUS devices are small, portable and easy to use and provide an invaluable tool to address a specific question in real-time at a patient’s bedside. With this increased use of PoCUS, there is an opportunity for midwives to undertake late pregnancy scans in all settings. Ultrasound is the gold standard for confirming fetal presentation, but midwives are not routinely trained in ultrasound in the UK. A retrospective study has shown that an ultrasound scan at 36 weeks’ gestation, either by a midwife using a PoCUS device or by a sonographer using a conventional ultrasound machine, reduces the incidence of undiagnosed breech and its associated outcomes^15^; however, there has not yet been a prospective study to assess the impact of PoCUS for this purpose. A survey has determined that midwives are keen to develop this new skill.^16^

The NIHR-funded Sono-breech study aims to determine the diagnostic accuracy and acceptability of midwife-performed PoCUS scans for fetal presentation at 36 weeks’ gestation. A health economics study will determine the cost-effectiveness of introducing this scan into routine antenatal care in the National Health Service (NHS).

## Methods and analysis

### Diagnostic accuracy

Eligibility criteria for pregnant women:

- Live, singleton pregnancy.
- 35^+0^–36^+6^ weeks’ gestation.
- Able to give valid, informed consent.
- Commitment to attend a second scan within 1 day.

At routine antenatal appointments, women will be informed about the study. Posters (see online supplemental appendix 1) will be placed in areas such as the antenatal clinic, and participant information sheets are available. The participant information sheet has been translated into five additional languages (Arabic, Hindi, Polish, Romanian and Urdu).

At an antenatal appointment between 35^+0^ and 36^+6^ weeks’ gestation, participants will be consented to take part in the study. Consent will be recorded using the REDCap database (see online supplemental appendix 2). The appointment may take place in the hospital or the community. Once consent has been obtained, the midwife will perform antenatal checks, including palpation for presentation, documenting their findings on the REDCap database. The midwife will then perform a scan using a handheld PoCUS device to check fetal presentation and viability, again documenting their findings in real-time on REDCap. Presentation is categorised as cephalic, breech, oblique/transverse or uncertain/unknown. For the purpose of the study, the categories will be dichotomised as cephalic or non-cephalic.

Before the end of the next day, the participant will then receive a second ultrasound scan by an ultrasound practitioner (eg, sonographer or obstetrician) using a conventional ultrasound machine to confirm the midwife’s findings. The second scan may be a planned routine scan or may be an additional scan specifically for the study. This scan will also include confirmation of viability and measurement of the deepest vertical pool of amniotic fluid.

If the participant is found to have a baby in cephalic presentation, there will be no change to their pathway of care. If the participant is found to have a baby in non-cephalic presentation, they will be referred to their local NHS Trust’s pathway for non-cephalic presentation at 36 weeks.

The handheld PoCUS devices being used in the study are the GE VScan Air (GE Healthcare Technologies**,** Chicago, Illinois, USA), Clarius C3 HD3 (Clarius Mobile Health, Burnaby, BC, Canada) and Philips Lumify (Philips Healthcare, Amsterdam, The Netherlands). For the purpose of the study, the devices are considered to have equivalent functionality and will not be compared; the purpose is to test the concept of handheld scanning rather than compare specific equipment. The ultrasound image display is either on a smartphone or an e-tablet.

### Training

Since midwives do not routinely receive ultrasound training in the UK, there was a need to upskill midwives before the start of the study. While training and competence have ultimately remained the responsibility of the local principal investigators, an optional training pathway was developed by the team at Imperial College London, comprising a 2-hour ultrasound simulation training session using Volutracer OPUS software, followed by a half-day observation session with a sonographer and completion of a competency log (10 fetal presentation scans, including one non-cephalic presentation performed under supervision) in clinical practice. Once midwives have completed the training pathway and attended a presentation for the site initiation visit that includes proportionate study-specific training on consent and Good Clinical Practice, they are deemed competent to scan as part of the Sono-breech study. Feedback from these simulation sessions has shown that midwives feel that simulation training is beneficial.^17^

### Acceptability

To ensure successful implementation of PoCUS in routine antenatal care, it must be acceptable to both the service users receiving the scan and the midwives performing the scan. Acceptability will be assessed using a framework evaluating healthcare interventions, which comprises seven constructs.^18^ 6 Sono-breech sites will be selected for the acceptability study to ensure representation across different settings. The acceptability study will involve interviews, focus groups and non-participant observation with women and midwives to explore what they think about the introduction of PoCUS into routine midwifery antenatal care.

#### Participants

Study participants, selected to ensure representation from those with both cephalic babies and breech babies, will be interviewed to gain their thoughts on the scan. Those who declined to have the scan but are happy to be interviewed will also be contacted to explore the reasons people may prefer not to have a PoCUS scan. They will be contacted between 6 and 12 weeks after birth and invited to take part in a 1-hour semistructured interview at a time and place (in person, online or via telephone) convenient to them. An interview schedule based on the theory of acceptability has been developed for the study. Women who take part in an interview are offered a £25 voucher for their time.

#### Midwives

Midwives from six sites, who are trained and performing the PoCUS scans for the study, will be invited to participate in focus groups and/or interviews. The focus groups and interviews will be semistructured and will follow a schedule based on the theory of acceptability.

Non-participant observation will be undertaken during PoCUS training and antenatal clinics. A member of the acceptability qualitative research team will observe a midwifery clinic and record observation notes, looking at verbal and physical interactions and behaviours. This will help identify potential facilitators and barriers to implementing PoCUS into midwifery training and routine antenatal care.

### Health economics

The Health Economics study will assess the cost-effectiveness of implementing the PoCUS scan at 36 weeks’ gestation into routine care compared with the current standard of care (abdominal palpation). This will involve analysis of maternal and neonatal outcomes (eg, cost of birth, cost of length of admission) as well as self-reported outcomes, via self-completed questionnaires sent to the participant 6 weeks post-delivery (Birth Experience Assessment Measure, developed by University College London and City St George’s, University of London) and Infant Quality of Life Instrument (IQI).

### Study timeline

Study set-up: up to 10 months. The study opened in August 2024, and recruitment will be open for 27 months, with projected completion in November 2026 and follow-up finishing by 12 weeks after the last participant has given birth.

## Study outcome measures

Note that for the purposes of this study, we will consider the primary outcome to be a non-cephalic presentation (breech+transverse/oblique).

### Primary outcome

Diagnostic accuracy (sensitivity and specificity) of midwife-conducted handheld PoCUS at 36 weeks for the detection of non-cephalic presentation compared with ‘gold standard’ conventional ultrasound in the same women.

### Secondary outcomes

(a) Acceptability to midwives and pregnant women of handheld PoCUS in the detection of non-cephalic presentation at term; (b) resource use, including mode of birth and neonatal intensive care unit (ICU)—reasons for and outcomes of admissions and length of stay obtained from patient records for up to 28 days (table 1); (c) evaluation of training requirements for midwives using handheld PoCUS for fetal presentation; (d) prevalence of undetected breech presentation in early labour in the cohort undergoing PoCUS compared with those not undergoing PoCUS; (e) Birth Experience Assessment Measure and (f) IQI.

**Table 1:** Maternal and neonatal outcomes collected from participants’ medical notes following delivery

Maternal outcomes	Neonatal outcomes
Maternal death	Neonatal death
Postpartum haemorrhage (blood loss>500 mL)	Apgar scores at birth: 1, 5 and 10 min
Blood transfusion	Umbilical cord gas pH level (arterial and venous) and base excess (if collected)
Maternal ICU admission	Admission of the neonate within 24 hours to (a) NICU; (b) SCBU and (c) transitional care
Operative complication during a caesarean delivery (ureter, bladder or bowel injury)	Neonatal trauma: bone fractures, skull haematoma, intraventricular or intracerebral haemorrhage, occurrence of brachial plexus injury, facial nerve palsy
Episiotomy	Length of neonatal admission
Perineal tears and obstetric anal sphincter injury	Jaundice
Intrapartum or immediate postpartum infection requiring treatment according to the sepsis protocol	
Postpartum complications: vaginal haematoma, venous thromboembolism, bowel obstruction	
Length of postnatal hospital inpatient admission	

ICU, intensive care unit; NICU, neonatal intensive care unit; SCBU, Special Care Baby Unit.

Secondary outcomes (b), (e) and (f) will be analysed as part of the economic evaluation to evaluate the cost-effectiveness of handheld PoCUS compared with standard care.

## Data collection

Data for the primary outcome and secondary outcomes (b), (d), (e) and (f) will be collected using a secure online database (REDCap). Participants will be anonymised and will receive a unique study number in the database. Their telephone number and email address will be collected and stored securely so that they can be contacted about the questionnaires±interviews after delivery. The interviews, observations and focus groups that take place for secondary outcome (a) will be recorded and transcribed; they will be stored securely and then deleted. The data for secondary outcome (c) will be recorded using self-completed paper feedback forms.

Primary outcome data will be collected at the time of the initial PoCUS scan and by the end of the next day following the initial scan. Data for secondary outcome (a) will be collected by the qualitative research team; acceptability to midwives will be assessed throughout the study and acceptability to women will be assessed between 6 and 12 weeks post-delivery. Secondary outcome data for outcomes (b) and (d) will be collected from the participant’s medical notes by a research midwife. Outcomes (e) and (f) will also be collected between 6 and 12 weeks post-delivery via self-completed questionnaires sent to the participant by email. Secondary outcome data for (c) will be collected following midwife ultrasound training sessions that took place in the set-up phase of the study, as well as from observations by the qualitative research team.

Additional anonymous aggregate birth data will be collected about all women who deliver at each site to determine the number of undiagnosed breech births in those who have not participated in the study.

### Data analysis

#### Diagnostic accuracy study analysis plan

Diagnostic accuracy of at least 90%, as determined by sensitivity and specificity, is required for the PoCUS scan to be considered clinically useful. Sensitivity is the proportion of true positives (correctly identified non-cephalic presentation), and specificity is the proportion of true negatives (correctly identified cephalic presentation). We conservatively estimate sensitivity at 95% based on a previous study, allowing estimation of diagnostic accuracy with a 5% margin of error (lower CI 90%).^19^

The minimum acceptable criterion for sensitivity and specificity is set at 0.9. Based on an expected sensitivity of 0.95 (with alpha=0.05 and power=0.90), at least 356 participants with non-cephalic presentation are required to ensure that the lower limit of the one-sided CI is at least 0.9. Assuming an expected specificity of 0.95, 371 participants with non-cephalic presentation are required to ensure that the lower limit of the one-sided CI is at least 0.9. The expected prevalence of non-cephalic presentation is 4.1%^20^; therefore, at least 8683 participants are required. The target for the study has been inflated by 10% to 9648 to account for issues such as missed second scans and withdrawal of consent.

Throughout the study, the prevalence of non-cephalic presentation and dropout rate will be monitored. If the true prevalence is higher than 4%, fewer participants may be needed overall. Recruitment will continue until 356 non-cephalic presentations have been identified by the sonographer scan with an accompanying PoCUS scan result.

Diagnostic measures of accuracy (sensitivity, specificity, positive predictive value and negative predictive value) will be reported with 95% CIs. We will use asymptotic normal distribution theory to calculate joint CIs for both sensitivity and specificity. We will calculate the 97.5% univariate CIs for the True Positive Fraction (sensitivity) and False Positive Fraction (1−specificity) and use these to calculate a rectangular region that represents the joint 95% CI for both sensitivity and specificity.^21 22^ Sensitivity analyses will compare diagnostic accuracy measures in the complete case population with imputed worst/best-case scenario measures. Separate sensitivity analyses will be carried out excluding participants not between 35^+0^ and 36^+6^ weeks of gestational age and women who have the handheld and confirmatory ultrasound scans more than 1 day apart.

Both diagnostic tests are expected to occur on the same day; however, the time interval between the PoCUS and sonographer scan will be summarised, and any ‘out-of-window’ assessments will be reported. We will also compare the primary outcome between in-house site midwife training and Sono-breech study midwife training.

Secondary outcomes include non-cephalic presentations that remain undiagnosed in labour, maternal and neonatal outcomes, modes of delivery and non-cephalic presentations missed by abdominal palpation. Continuous outcomes will be summarised as means with SD if normally distributed or medians with IQR if data are skewed. Binary and categorical variables will be summarised as frequencies and percentages.

All results and any adverse events (AEs) will be reported according to the Standards for Reporting of Diagnostic Accuracy Studies.

#### Acceptability study analysis plan

Interviews, focus groups and observations will be transcribed and coded using the qualitative analysis software NVivo. The analysis approach will employ a mixture of deductive and inductive reasoning techniques. This initial analysis will then be mapped against the theoretical framework of acceptability,^18^ generating findings on the willingness to change practice, any improvements to maternity care experiences and study protocol fidelity. Inductive, thematic analysis will produce cross-cutting themes relevant to the acceptability and implementation of PoCUS. The results of the analysis will be reported using Standards for Reporting Qualitative Research.

#### Health economics analysis plan

The following costs will be calculated:

- Annuitised cost of handheld PoCUS scan per pregnant woman (based on study data, cost of equipment, maintenance costs, lifetime of equipment and midwife training costs).
- Cost of additional time needed in antenatal appointments to complete PoCUS scan (based on cost per minute of midwives’ time).
- Cost per pregnant woman of the care pathway for fetal position at 36 weeks and fetal presentation at delivery (based on study data, cost of delivery, eg, emergency vs planned caesarean, vaginal birth with and without instruments).

The IQI and Birth Experience Assessment Measurement questionnaires, plus their respective preference-based tariffs, will be used to capture the preference-based quality of life of newborns and mothers, respectively.

We will report descriptive statistics for cost-based and preference-based outcomes for non-cephalic versus cephalic presentations. A decision model will be developed to incorporate the information from the study on handheld PoCUS sensitivity and specificity. This will include an estimation of the reduction in emergency caesareans and other related injuries following correct identification of presentation-based study data with a comparator for current practice based on data from the literature. The primary analysis will be from a maternity and neonatal perspective, with a time horizon from 36 weeks prenatal until 6–12 weeks postnatal. A secondary analysis will take a lifetime horizon, incorporating lifetime quality-adjusted life year losses and costs from maternal and infant mortality from a health and personal social services perspective.

Uncertainty, including cost-effectiveness acceptability curves and cost-effectiveness planes, will be calculated using probabilistic sensitivity analysis. Costs and outcomes after 1 year will be discounted in line with National Institute for Health and Care Excellence (NICE) guidance.^23^ One-way, two-way and multi-way sensitivity analyses will be conducted for any key assumptions made. Missing data are most likely to be minimal for the delivery resource use, but high for self-reported preference-based quality of life. Descriptive statistics will be reported for complete cases only, with sensitivity analyses looking at the use of multiple imputation to impute missing data based on predictors of missingness and assuming that data are missing at random.

## Public and patient involvement and engagement

There has been public and patient involvement and engagement (PPIE) throughout the study. The study was initially designed with input from The Bridge, a local community PPIE group led by the lead research midwife from the Women’s Health Research Centre at Imperial College London, UK.

During study set-up, a study-specific PPIE panel was formed. The PPIE panel is led by the founder and chief executive of Cysters,^24^ a community-led charity that works to promote the health, education and well-being of mothers, particularly those from marginalised backgrounds. The panel also includes representation from the charities Tommy’s^25^ (a pregnancy and baby charity), Sands^26^ (a pregnancy and baby loss charity) and Baby Lifeline^27^ (a maternity safety charity), as well as doula representatives from the Latin doula community and the founder of Doulas Decolonising, all of whom are well known within the community for their outreach work. The head of research and development from Baby Lifeline is also on the study steering committee. There are several service users with lived experiences of breech presentation at term. There are representatives from maternity community networks, such as Imperial Maternity Voices Partnership, as well as those representing ethnic minorities, neurodiverse and gender-diverse populations.

Cysters joined the project after the initial PPIE panel had been recruited and undertook additional targeted outreach through their established community networks to recruit a more diverse group of PPIE members. This resulted in the successful engagement of individuals from marginalised backgrounds, many of whom had not previously participated in PPIE activities. Cysters conducted one-to-one meetings with these members to explain their roles in the research, build understanding of the study and ensure they felt confident and supported to contribute meaningfully. In addition to regular group meetings, Cysters provided ongoing individual support sessions to address questions, strengthen confidence in speaking about the research and support members’ personal development, thereby enabling equitable participation and reducing barriers to involvement.

The panel has met through the set-up and early phases of the study, advising on the drafting of public-facing and participant-facing materials, including the participant information sheet, consent form, health economics questionnaires, Sono-breech website (https://www.sonobreech.uk/) and social media. Feedback has been incorporated into these materials to ensure they are appropriate and accessible.

### Adverse events

As the study is deemed low-risk, AEs are only collected when they occur while the PoCUS or conventional ultrasound scan is being delivered. AE is defined as any untoward medical occurrence in a patient or clinical study subject. Serious AE (SAE) is defined as any untoward medical occurrence or effect that results in death, is life-threatening, requires hospitalisation or prolongation of existing inpatient hospitalisation, results in persistent or significant disability or incapacity or is a congenital anomaly or birth defect. We do not expect any AEs in this study.

AEs should be reported in the participant’s medical notes. Any SAEs should be reported to the chief investigator within 24 hours, and related and unexpected SAEs should also be reported to the Research Ethics Committee (REC) within 15 days.

### Ethics

The study received a favourable ethical opinion from the West Midlands–South Birmingham REC and was approved by the Health Research Authority and Health and Care Research Wales. Each study site (NHS Trust) must confirm capacity and capability to deliver the study before any study activities can take place.

All those involved in delivering the study, including midwives and sonographers, are required to attend a presentation for the site initiation visit that includes details about the study and study-specific information related to taking consent for research, with proportionate Good Clinical Practice training included.^28^ All staff involved in delivering the study are required to sign the study delegation log and be suitably qualified to perform their roles in the study by completing the study integrated training log.

The study is registered under ISRCTN11748052.

### Dissemination plan

While the study is ongoing, updates will be available to the public via the study website (www.sonobreech.uk) and social media. The results of this study will be published in high-impact scientific journals. A publication policy will ensure efficient and transparent contributions to publication.

## Supplementary material

10.1136/bmjopen-2026-122247 online supplemental file 1

10.1136/bmjopen-2026-122247 online supplemental file 2

## References

[1] Wastlund D, Moraitis AA, Dacey A (2019). Screening for breech presentation using universal late-pregnancy ultrasonography: A prospective cohort study and cost effectiveness analysis. PLoS Med.

[2] Kaneti H, Rosen D, Markov S (2000). Intrapartum external cephalic version of footling-breech presentation. Acta Obstet Gynecol Scand.

[3] Morris SE, Sundin D, Geraghty S (2022). Women’s experiences of breech birth decision making: An integrated review. *Eur J Midwifery*.

[4] Furtado V, Kitson-Reynolds E (2021). Changing the tide: influencing factors for midwives on vaginal breech birth skill acquisition. Br J Midwifery.

[5] (2017). Management of Breech Presentation. *BJOG*.

[6] Berhan Y, Haileamlak A (2016). The risks of planned vaginal breech delivery versus planned caesarean section for term breech birth: a meta-analysis including observational studies. BJOG.

[7] Ekéus C, Norman M, Åberg K (2019). Vaginal breech delivery at term and neonatal morbidity and mortality - a population-based cohort study in Sweden. J Matern Fetal Neonatal Med.

[8] Fernández-Carrasco FJ, Cristóbal-Cañadas D, Gómez-Salgado J (2022). Maternal and fetal risks of planned vaginal breech delivery vs planned caesarean section for term breech birth: A systematic review and meta-analysis. J Glob Health.

[9] (2017). Magro five years of cerebral palsy claims. a thematic review of nhs resolution data.

[10] Chambers (2017). £37 million awarded to young boy left brain damaged at birth.

[11] Webb R, Ayers S, Bogaerts A (2021). When birth is not as expected: a systematic review of the impact of a mismatch between expectations and experiences. BMC Pregnancy Childbirth.

[12] Whitson MR, Mayo PH (2016). Ultrasonography in the emergency department. Crit Care.

[13] Johri AM, Glass C, Hill B (2023). The Evolution of Cardiovascular Ultrasound: A Review of Cardiac Point-of-Care Ultrasound (POCUS) Across Specialties. Am J Med.

[14] Shekhar AC, Blumen I (2021). A narrative review on the use of ultrasonography in critical care transport: is POCUS hocus?. Trends Anaesth Crit Care.

[15] Knights S, Prasad S, Kalafat E (2023). Impact of point-of-care ultrasound and routine third trimester ultrasound on undiagnosed breech presentation and perinatal outcomes: An observational multicentre cohort study. PLoS Med.

[16] Prasad S, Poljak B, Richens Y (2024). Training midwives to determine fetal presentation using a handheld portable ultrasound device-need of the hour?. *AJOG Glob Rep*.

[17] Thomas J SA, Kovalenko M, Clark A (N.d). Implementation and Evaluation of Ultrasound Simulation Training for Midwives: Insights from the Sono-Breech Study. Br J Midwifery.

[18] Sekhon M, Cartwright M, Francis JJ (2017). Acceptability of healthcare interventions: an overview of reviews and development of a theoretical framework. BMC Health Serv Res.

[19] Galjaard S, Baeck S, Ameye L (2014). Use of a pocket-sized ultrasound machine (PUM) for routine examinations in the third trimester of pregnancy. *Ultrasound Obstet Gynecol*.

[20] Impey LWM, Griffiths M PL, on behalf of the Royal College of Obstetricians and Gynaecologists (2017). Management of Breech Presentation. BJOG.

[21] Korevaar DA, Gopalakrishna G, Cohen JF (2019). Targeted test evaluation: a framework for designing diagnostic accuracy studies with clear study hypotheses. Diagn Progn Res.

[22] MS P (2003). The Statistical Evaluation of Medical Tests for Classification and Prediction.

[23] National Institute for Health and Care Intelligence (NICE) (2025). NICE health technology evaluations: the manual.

[24] Cysters. https://cysters.org.

[25] Tommy’s T The pregnancy and baby charity. https://www.tommys.org.

[26] SANDS S Saving babies’ lives. https://www.sands.org.uk.

[27] Lifeline B Baby lifeline, the mother and baby charity. https://www.babylifeline.org.uk.

[28] HRA M (2020). Devolved administrations for northern ireland, scotland and wales. joint statement on the application of good clinical practice to training for researchers.

