# Supplementary material for: Diagnostic accuracy of handheld ultrasound, performed in community and hospital settings at 36 weeks to determine fetal presentation: the Sono-breech study protocol
Source: BMJ Open. 2026 Sep 23;16(9):e122247. doi: 10.1136/bmjopen-2026-122247 (PMC13629926; doi:10.1136/bmjopen-2026-122247)
Supplement: Supplementary file 1 [file bmjopen-16-9-s001.pdf]

SONO-BREECH

IMPERIAL

FUNDED BY NIHR | National Institute for Health and Care Research

Cardiff University | Prifysgol Caerdydd — Centre for Trials Research | Canolfan Ymchwil Treialon

Sono-Breech

research study

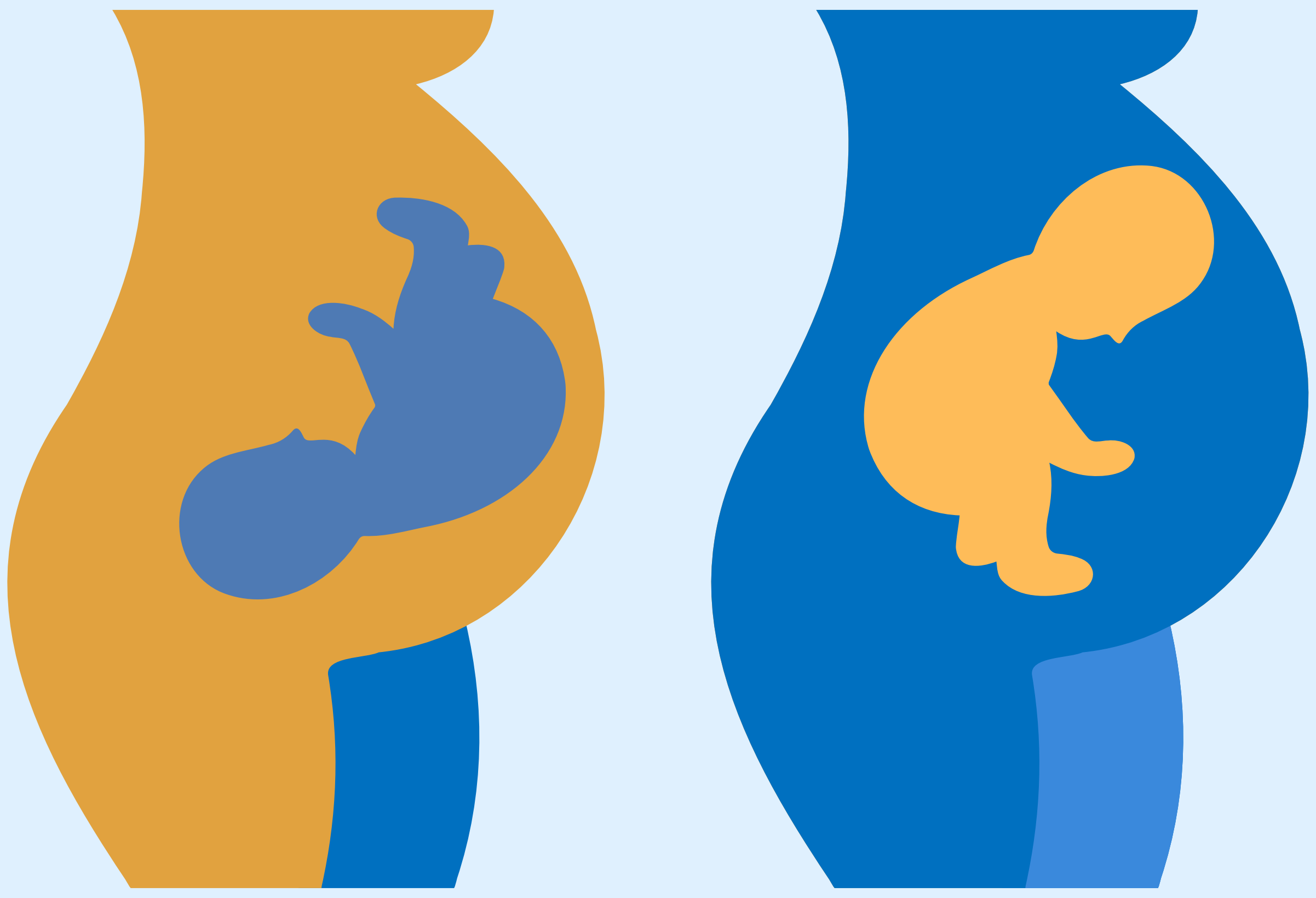

Are you in your third trimester and interested in research?

We want to know if midwives can accurately detect breech presentation at 36-weeks using handheld ultrasound machines

For more information, scan the QR code, speak to your midwife or email sono-breech@imperial.ac.uk

Instagram: @sonobreech

X: @sonobreech

Participant poster V1.2 04Jun2025
