# Supplementary material for: Diagnostic accuracy of handheld ultrasound, performed in community and hospital settings at 36 weeks to determine fetal presentation: the Sono-breech study protocol
Source: BMJ Open. 2026 Sep 23;16(9):e122247. doi: 10.1136/bmjopen-2026-122247 (PMC13629926; doi:10.1136/bmjopen-2026-122247)
Supplement: Supplementary file 2 [file bmjopen-2026-122247.reviewer_comments.pdf]

PEER REVIEW HISTORY

BMJ Open publishes all reviews undertaken for accepted manuscripts. Reviewers are asked to complete a checklist review form and are provided with free text boxes to elaborate on their assessment. These free text comments are reproduced below.

ARTICLE DETAILS

Title (Provisional)

Diagnostic accuracy of handheld ultrasound at 36-weeks to determine fetal presentation: the Sono-breech study protocol

Authors

Thomas, Jessica; Owen-Jones, Eleri; Kovalenko, Mariya; Clark, Anna; Usman, Sana; Beattie-Jones, Suzanne; Thornton, Danielle; Banerjee, Jayanta; Heera, Neelam; Scamell, Mandie; Yuill, Cassandra; Townson, Julia; Hunter, Rachael; Condie, Jennifer; Playle, Rebecca; Bhide, Amarnath; Lees, Christoph

---

This article was not externally reviewed at BMJ Open. Protocols that have been independently assessed prior to submission to BMJ Open are usually fast-tracked to publication on the grounds that further substantial changes will not be possible. This independent assessment will usually be external, independent review for both a substantial grant award from a non-commercial or government funder and ethics approval.
